# High-density lipoprotein proteome dynamics in human endotoxemia

**DOI:** 10.1186/1477-5956-9-34

**Published:** 2011-06-28

**Authors:** Johannes HM Levels, Pierre Geurts, Helen Karlsson, Raphaël Marée, Stefan Ljunggren, Louise Fornander, Louis Wehenkel, Mats Lindahl, Erik SG Stroes, Jan A Kuivenhoven, Joost CM Meijers

**Affiliations:** 1 Department of Experimental Vascular Medicine, Academic Medical Center, University of Amsterdam, Amsterdam, the Netherlands; 2 Department of Electrical Engineering and Computer Science & GIGA-research, University of Liège, Belgium; 3 Occupational and Environmental Medicine, Heart-Medical Centre, County Council of Östergötland, Department of Clinical and Experimental Medicine, Linköping University, Sweden; 4 GIGA Bioinformatics Platform, University of Liège, Belgium; 5 Occupational and Environmental Medicine, Department of Clinical and Experimental Medicine, Faculty of Health Sciences, Linköping University, Sweden; 6 Department of Vascular Medicine, Academic Medical Center, University of Amsterdam, Amsterdam, the Netherlands

## Abstract

**Background:**

A large variety of proteins involved in inflammation, coagulation, lipid-oxidation and lipid metabolism have been associated with high-density lipoprotein (HDL) and it is anticipated that changes in the HDL proteome have implications for the multiple functions of HDL. Here, SELDI-TOF mass spectrometry (MS) was used to study the dynamic changes of HDL protein composition in a human experimental low-dose endotoxemia model. Ten healthy men with low HDL cholesterol (0.7+/-0.1 mmol/L) and 10 men with high HDL cholesterol levels (1.9+/-0.4 mmol/L) were challenged with endotoxin (LPS) intravenously (1 ng/kg bodyweight). We previously showed that subjects with low HDL cholesterol are more susceptible to an inflammatory challenge. The current study tested the hypothesis that this discrepancy may be related to differences in the HDL proteome.

**Results:**

Plasma drawn at 7 time-points over a 24 hour time period after LPS challenge was used for direct capture of HDL using antibodies against apolipoprotein A-I followed by subsequent SELDI-TOF MS profiling. Upon LPS administration, profound changes in 21 markers (adjusted p-value < 0.05) were observed in the proteome in both study groups. These changes were observed 1 hour after LPS infusion and sustained up to 24 hours, but unexpectedly were not different between the 2 study groups. Hierarchical clustering of the protein spectra at all time points of all individuals revealed 3 distinct clusters, which were largely independent of baseline HDL cholesterol levels but correlated with paraoxonase 1 activity. The acute phase protein serum amyloid A-1/2 (SAA-1/2) was clearly upregulated after LPS infusion in both groups and comprised both native and N-terminal truncated variants that were identified by two-dimensional gel electrophoresis and mass spectrometry. Individuals of one of the clusters were distinguished by a lower SAA-1/2 response after LPS challenge and a delayed time-response of the truncated variants.

**Conclusions:**

This study shows that the semi-quantitative differences in the HDL proteome as assessed by SELDI-TOF MS cannot explain why subjects with low HDL cholesterol are more susceptible to a challenge with LPS than those with high HDL cholesterol. Instead the results indicate that hierarchical clustering could be useful to predict HDL functionality in acute phase responses towards LPS.

## Introduction

High-density Lipoprotein (HDL) cholesterol levels are inversely associated with a risk for cardiovascular disease [1]. LDL cholesterol lowering drugs have been proven to be successful, however up to 65% of cardiovascular death cannot be prevented which explains the strong interest in HDL increasing strategies [2].

Intensive research over the past decade has elucidated the involvement of HDL in multiple pathways in atherogenesis. Besides hyper- and hypolipidemia, oxidation of lipoproteins and coagulation and fibrinolysis [3], Iinflammation is suggested to be one of the fields in which HDL and HDL-associated proteins [4] affect the course of acute infection. As a result, the consequential systemic inflammatory response may have a causal impact on atherosclerosis [5].

Proteomic biomarker search has been recently applied to HDL to achieve more insight into the functions and protein composition of this lipoprotein class [6-8]. Assessment of 1-dimensional electrophoresis (DE) and 2-DE-matrix-assisted laser desorption ionisation-time of flight, Isotope-coded Affinity Tag and Western-blot analysis has led to the identification of 56 HDL-associated proteins [9,10]. Importantly, Vaisar *et. al. *identified 42 HDL-associated proteins that differed substantially in amount between HDL from CAD patients and controls [11]. Serial sample analysis with the above mentioned approaches are labor intensive which prompted us establish a high-throughput HDL profiling technique [9]. Moreover, serial profiling of the HDL proteome might be a welcome addition to reveal information about the dynamic processes that occur compared to single "snapshot" approaches. The current study focuses on the HDL protein dynamics by means of SELDI-TOF MS after an endotoxin intervention in healthy volunteers.

## Methods

### Study Participants

Healthy male subjects with plasma HDL cholesterol levels < 10th percentile (low HDL group, n = 10) and healthy age-matched male subjects with plasma HDL cholesterol levels > 90th percentile (high HDL group, n = 10), were recruited for endotoxin challenge [12]. In addition, normolipemic males (n = 4; HDL cholesterol levels 1.4 +/- 0.3 mM) were recruited as controls for a vehicle bolus. Written informed consent was obtained from all participants and the study protocol was approved by the institutional review board of the Academic Medical Center.

### Study Design

The study was performed as described [12]. In brief, study participants were required to refrain from alcohol and caffeine-containing beverages at least 24 hours before the study. On the morning of the study day at 7:30 AM after an overnight fast, study participants were admitted to the research unit. At 7.45 AM a catheter was inserted in an antecubital vein of each arm. At 8.00 AM (time [t] 0), blood was drawn for baseline measurements. Subsequently, subjects received a bolus infusion of 1 ng/kg body weight of endotoxin (*Escherichia coli *lipopolysaccharide, catalog number 1235503, lot G2B274; United States Pharmacopeial Convention Inc, Rockville, MD) in the antecubital vein of the contralateral arm. Healthy controls received a vehicle bolus to control for any diurnal effects. Blood samples were collected at t = 0, 1, 2.5, 4, 6, and 8 hours after endotoxin challenge. The next morning at 8.00 AM, 24 hours after endotoxin infusion, study participants returned after an overnight fast for a final blood withdrawal.

### Blood collection

Blood was collected in EDTA or heparin, kept on ice, and centrifuged at 2000*g *for 20 minutes at 4°C. Plasma was snap-frozen and stored at -80°C until analysis.

### Inflammatory marker protein measurements

Serum amyloid A (SAA) ELISA was carried out using a kit from Anogen biotech Laboratories (Mississauga, Ontario, Canada). PON-1 activity measurements were performed in heparinized plasma as described previously [13].

### Sample preparation and HDL capture

SELDI-TOF MS analysis was performed as described [9]. Complete time series of plasma of each participant was used for analyses on separate spots on PS20 chips (in triplicate). In short, native HDL was directly captured from 100 μL diluted EDTA plasma (1:2 diluted with Tris Buffered Saline pH 7.4) which was incubated on chips coated with antibodies against apo A-I for 2 hours at room temperature on a horizontal shaker (600 rpm). The chips were subsequently washed 4 times with TBS for 10 minutes, followed by a 5 minute TBS-Tween (0.005%) rinse and a final wash step with Hepes solution (5 mM). All spots were allowed to dry and subsequently 1.2 μL sinapinic acid (10 mg/ml in 50/49.9/0.1% acetonitril/H_2_O/TFA) was applied to each spot.

### SELDI-TOF analysis and data preprocessing

Spectra were recorded at two laser intensities of 203 to 210 relative units and the focus mass was set to 28 kDa. Spectra were generated with approximately 100 shots at 13 positions per SELDI spot. Analysis was carried out using a PBS IIc protein chip reader (Ciphergen Biosystems, Fremont, CA, USA) using an automated data collection protocol of the Protein-Chip Software (version 3.1.1.). Data were collected up to 150,000 kDa. Calibration was performed using a protein calibration chip (Bio-Rad). All spectra were automatically corrected for baseline values and spot to spot correction was assessed by the Ciphergen protein chip software. Finally, since the HDL-capture design was based on a 100% chip saturation with HDL particles, all spectra were normalized on apo A-I signal.

### HDL isolation for two-dimensional gel electrophoresis (2-DE), protein quantification and identification

HDL isolation of six subjects, three representing the high HDL and three the low HDL groups, was performed before (t = 0) and after (t = 8) LPS challenge as described previously [6]. In brief, 5 mL of EDTA-plasma, adjusted to a density of 1.24 g/mL with solid KBr (0.3816 g/mL) was layered in the bottom of a centrifuge tube. The plasma fraction was then overlaid with KBr/phosphate solution (0.083 4 g/mL, density 1.063 g/mL). Ultracentrifugation was performed in a Beckman XL-90 equipped with a Ti 70 rotor at 290 000*g for 4 hours at 15°C. The fractions corresponding to HDL2 and HDL3 were collected with a syringe and a second ultracentrifugation step (KBr/phosphate solution, density of 1.24 g/mL) was performed for 2 hours to eliminate the presence of plasma contaminants. HDL proteins were desalted using PD-10 columns and protein concentration was measured using Bio-Rad protein assay. 2-DE was performed using IPGphor and Multiphor, Amersham Biosciences. HDL proteins (300 μg) were applied by in gel rehydration for 12 hours at low voltage (30 V) in pH 3-10NL IPGs strips. Proteins were then focused at 53000 Vh at max voltage of 8000 V. The second dimension was performed by transferring the proteins to a homogenous gel (T = 14%, C = 1.5%) running at 40-800 V, 10°C, 20-40 mA overnight. Separated proteins were stained by Sypro Ruby (Molecular Probes, USA). Images of the protein patterns were analyzed by a CCD camera in a UV-scanning illumination mode (Flour-S Multi-Imager, Bio-Rad, USA) combined with a computerized imaging 12-bit system (PDQuest 2-D gel analysis software, Bio-Rad).

Proteins were quantified as fluorescence intensity (counts) per total fluorescence on the 2-D gels, expressed in %. Proteins were identified by peptide mass fingerprinting of tryptic peptides using Matrix Assisted Laser Desorption/Ionization Time of Flight MS (MALDI TOF MS) (Voyager DE Pro, Applied Biosystems, USA) prior to database searches as described previously.

### Bioinformatics and statistics

Prior to statistical analysis all generated spectra were preprocessed as described before [14]. In brief, each individual m/z spectrum was divided into non-overlapping intervals whose sizes are increasing proportionally with the mass over charge (m/z) values. The size of an interval starting at 3000 (m/z) is computed as m.r with r fixed at 0.5% in all our results below. The intensity associated with each interval was taken as the sum of the intensities over the interval. Discretisation of the m/z axis leads to a reduction of the number of variables down to 704 m/z intervals.

Subsequently, a statistical test was performed for removal of the m/z intervals over which the intensity did not significantly change over time. For that purpose, the non-parametric Kruskal-Wallis one-way analysis of variance method was used for testing the equality of population medians among the 7 time points; 104 m/z intervals at the low laser intensity and 77 m/z intervals at the high laser intensity showed a p-value lower than 0.05. Raw p-values were then adjusted for multiple testing using Benjamini and Hochberg's method. This reduced the number of significant intervals down to 21 and 14, respectively. Since the high laser intensity showed less sensitivity, we restricted our analysis to the low laser intensity.

Hierarchical clustering was performed using, for each individual, the mean (of the triplicates) dynamics of all 21 significant markers. Hierarchical clustering was computed using an agglomerative approach with complete linkage and one minus the correlation (1-r) as a distance measure. All analyses were performed with Matlab^® ^(The MathWorks Inc., Natick, MA, USA).

## Results

We previously showed that infusion of endotoxin at 1 ng/kg bodyweight in healthy human volunteers with low and high HDL cholesterol levels did not affect changes in the absolute plasma levels of HDL cholesterol and apo A-I (Additional file 1, Figure S1) [12]. In the same study, it was shown that individuals with lower HDL cholesterol levels were more susceptible to this inflammatory stimulus based on leukocyte response, cytokine response and clinical symptoms compared to those with higher HDL cholesterol levels. The current study in which a subset (Table 1) of the original study cohort was used focuses on the changes in the HDL proteome.

**Table 1:** Characteristics of the study subjects

	Unit	Low HDL (n = 10)	High HDL (n = 10)
Age	(Years)	30 ± 17	34 ± 14

BMI	(kg/m^2^)	23.1 ± 5.7	22.4 ± 4.7

HDL-chol	(mM)	0.78 ± 0.14 *	1.98 ± 0.41 *

LDL-chol	(mM)	2.50 ± 0.55	2.62 ± 0.84

Apo A-I	(g/L)	1.0 ± 0.2 *	1.7 ± 0.2 *

Apo B	(g/L)	1.1 ± 0.2	1.0 ± 0.2

hsCRP	(g/L)	3.8 ± 4.8	1.5 ± 1.5

Total Chol	(mM)	3.84 ± 0.71	5.20 ± 1.10

IMT	(mm)	0.59 ± 0.11	0.54 ± 0.05

BMI, body mass index; HDL-chol, high-density lipoprotein cholesterol; LDL-chol, low-density lipoprotein cholesterol; Apo A-I indicates apolipoprotein A-I; apo B, apolipoprotein B; hsCRP, high-sensitivity C-reactive protein; IMT, intima-media thickness. All data are expressed as mean ± SD. * indicates P-value < 0.001 (Independent Student *t-*test).

### Serial HDL proteome profiling

**Figure 1:**
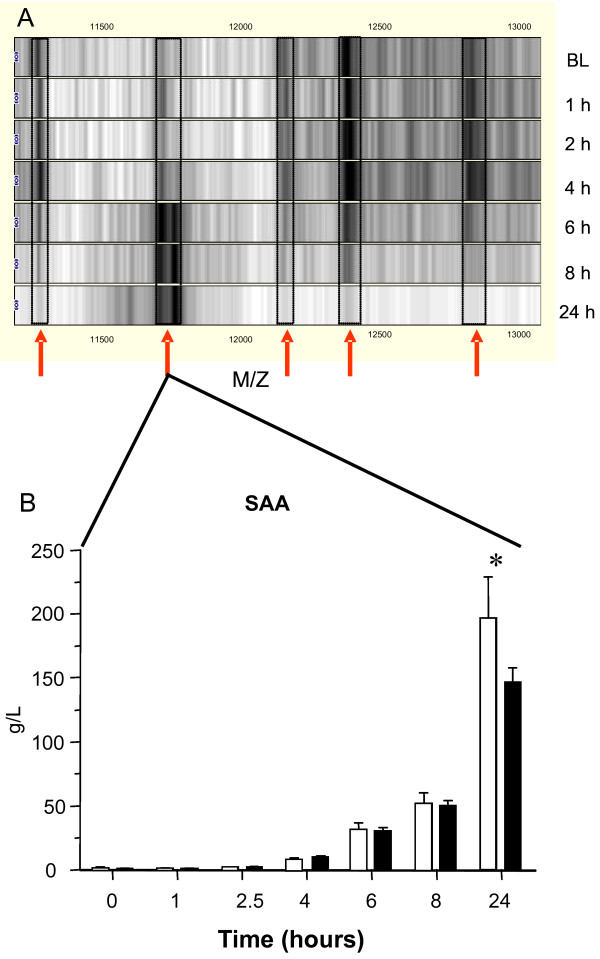
**Changes  in HDL composition upon LPS challenge.A**. Virtual gel-views representing the HDL composition after SELDI-TOF MS analysis. Depicted are profiles from on-chip captured HDL from baseline (BL) to 24 hours after endotoxin challenge. All spectra were normalized on total ion current and baseline corrected and subsequently translated to the "virtual gel-view" output format. The arrows indicate those markers which demonstrated a significant dynamic change over time (adjusted p < 0.05) after LPS challenge. **B. **SAA response in healthy individuals after LPS challenge. SAA levels, of the low (white bars) and high (black bars) HDL cholesterol groups, as determined by ELISA, are depicted. The data represent mean ± SD. * indicates statistical significant difference between the low and high cholesterol HDL group (p < 0.05). From t = 4 hours and all following timepoints a statistical significant difference compared to baseline condition was reached (p < 0.001).

For each time point, plasma HDL was directly captured using anti apo A-I antibodies on PS-20 protein-chips after which protein spectra were generated by SELDI-TOF mass spectrometry [9]. Figure 1A gives a representative overview of the HDL proteome changes over time following LPS administration of which serum amyloid-1 (SAA-1) could be confirmed with ELISA (Figure 1B). To study the dynamic changes in more detail, each spectrum was divided in 704 m/z marker intervals.

**Figure 2:**
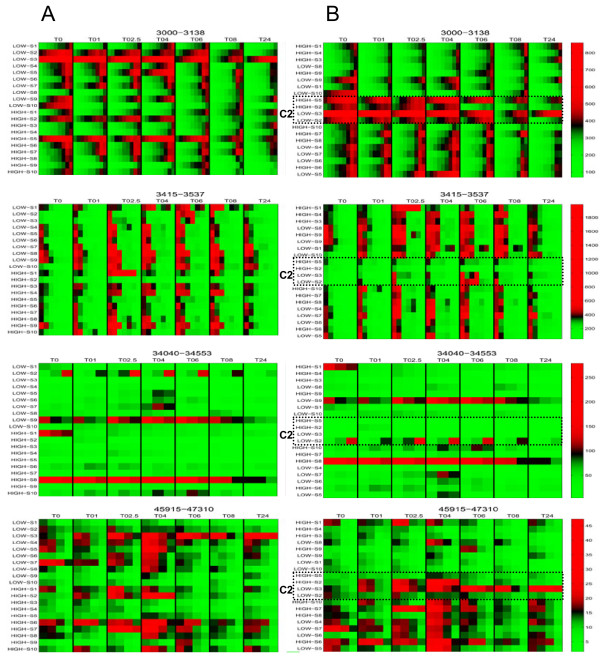
**Heat map overviews of the mass/charge (m/z) regions of the relevant changes in HDL dynamics upon LPS challenge**. Depicted are the mass intensity ranges (as indicated per heatmap) according to baseline HDL cholesterol level (A, left panels) and after clustering analysis (B, right panels) from baseline to 24 hours after LPS infusion. In each plot, the color scale has been centered at the mid-point between the 2% and 98% quantiles. Color values below the 2% and above the 98% quantiles have been saturated. C2; cluster 2 indicated by the rectangle.

**Figure 3:**
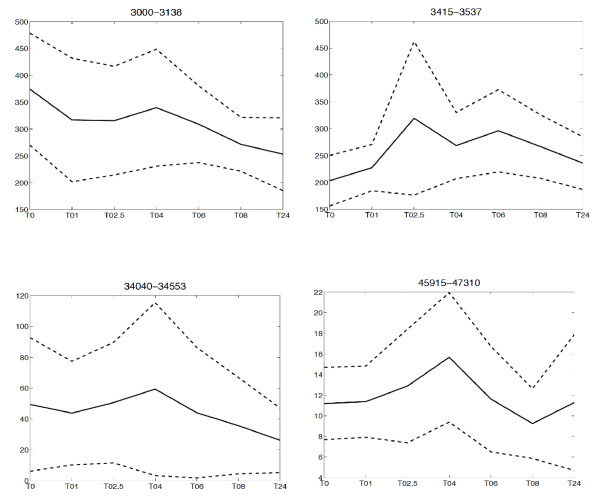
**Plots representing the dynamics of the intensities of the intervals of interest averaged over all individuals**. The dashed lines indicate plus/minus one standard deviation. In the x-axis the time course is indicated whereas the y-axis represents the total mean peak area.

In a next step, we generated heatmaps of the marker regions with the most prominent changes (Left panels of Figure 2). In each heatmap, the relative intensities from all timepoints of the indicated m/z region in the HDL spectrum are presented. The most significant changes were found in the low mass region (up to 20 kDa, Additional file 2, Table S1). Different types of dynamics, as shown in the plots of the dynamics in figure 3, were observed such as a gradual decrease in the 3000-3138 m/z marker interval, and a rise and fall with a maximum at t = 2.5 hours after endotoxin infusion in the 3450-3537 m/z marker interval. In the higher mass regions, a gradual decrease in marker intensity could be observed in the 34040-34553 m/z marker interval whereas in the 45915-47310 m/z marker interval a rise and fall with a maximum at t = 4 hours was seen (Figure 3).

In further analyses, we pooled the data of both study groups and found that 104 of the 704 m/z intervals changed significantly (p < 0.05) over the time course of the experiment, among which 21 remained significant after multiple testing correction (Additional file 2, Table S1). In the 4 control subjects, infused with vehicle, none of these 104 marker intervals changed significantly over the 24 hour time period indicating that diurnal rhythm was unlikely to cause the observed changes (Additional file 2, Table S1, right column).

Unexpectedly, we did not detect distinct differences in HDL proteome dynamics between the two study groups (Figure 2, left panels).

### Clustering analysis

**Figure 4:**
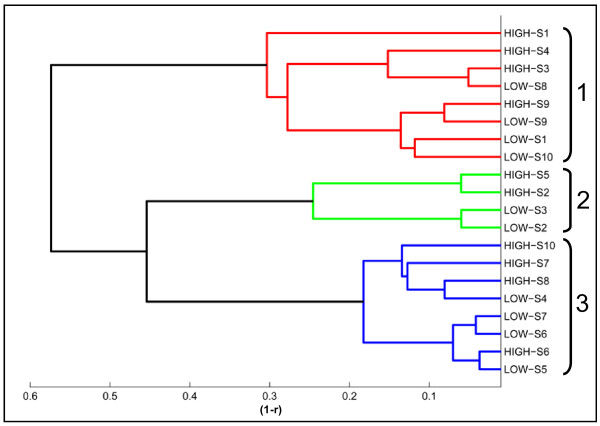
**Dendrogram representing the overall clustering over the 24 hour time period**. After correction for multiple measurements, 21 of the 104 markers of interest (p < 0.05) were included in the analysis. Three distinct clusters can be appreciated containing subjects from both the high and low HDL groups. The axis indicates one minus the correlation coefficient (1-r).

Since we did not observe a relation between baseline HDL cholesterol levels and HDL protein dynamics after endotoxin challenge, we investigated whether it was possible to identify individuals (in both groups) that could be clustered using the available proteomics data. By hierarchical clustering, the mean dynamics of all 21 relevant markers intervals was computed using an agglomerative approach with complete linkage and one minus the correlation as a distance measure. The right panels of Figure 2 show the clustering of the individuals upon LPS challenge of each of the given molecular mass intervals. The dendrogram in Figure 4 shows that three distinct clusters of individuals could be distinguished. Each cluster comprised individuals with both low and high HDL.

### Serum amyloid A-1/2 and paraoxonase as markers of HDL under inflammatory conditions

Since both serum amyloid A-1/2 (SAA-1/2) and paraoxonase-1 (PON1) may determine the function of HDL, we tested the hypothesis whether the LPS-challenged individuals displayed a cluster or group dependent distribution for both parameters. SAA-1/2 levels as determined by ELISA show that the LPS administration resulted in a similar increase in SAA-1/2 levels in both groups up till t = 8 (Figure 1B). At t = 24, however, the increase of SAA-1/2 in the low HDL group was slightly stronger than that in the high HDL group (p < 0.05).

**Figure 5:**
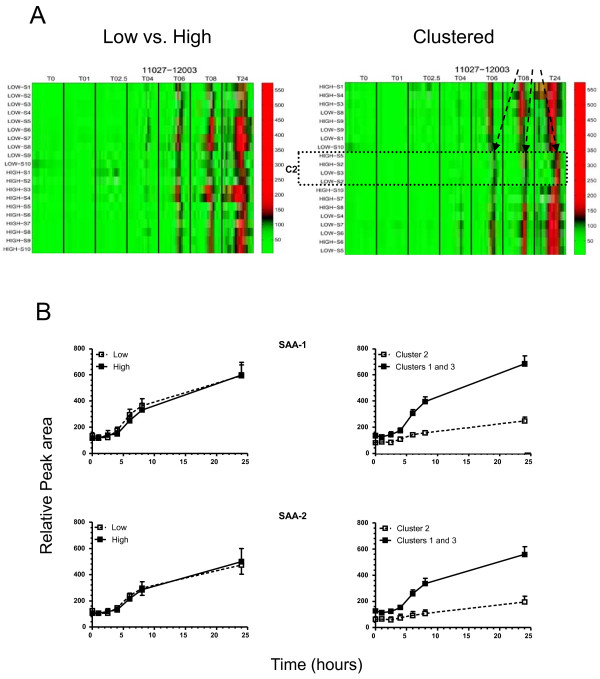
**Heat-map overviews of the (mean) dynamics in the (m/z) region corresponding with SAA**. Depicted are the mass intensity ranges (as indicated per heatmap) according to baseline HDL cholesterol level (left upper panel) and after clustering analysis (right upper panel) from baseline to 24 hours after LPS infusion. In each plot, the color scale has been centered at the mid-point between the 2% and 98% quantiles. Color values below the 2% and above the 98% quantiles have been saturated. The arrows indicate the larger molecular weight SAA-1 isoform specific for cluster 2 (C2). The lower graphs show the total SAA signal in time of SAA-1 and SAA-2 between the low and high HDL groups (middle left and lower left graphs) and the clusters 1/3 combined and cluster 2 (middle right and lower right graph respectively (p < 0.001)).

**Figure 6:**
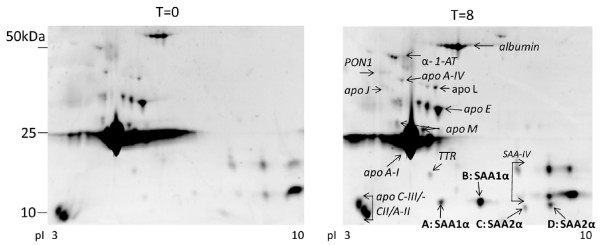
**Comparison of the HDL protein pattern from one subject before (t = 0) and after (t = 8) LPS exposure**. HDL was isolated from plasma by density-gradient ultracentrifugation. Proteins (300 μg) were separated with 2-DE and detected by Sypro Ruby staining. The proteins were quantified as fluorescence intensity per total protein fluorescence. The four SAA isoforms, as identified by mass spectrometry (see Figure 7) shown in the t = 8 h image represent: (A) SAA-1α-desArg, -desArgSer and -desArgSerPhePhe; (B) SAA-1α; (C) SAA-2α-desArg, -desArgSer and -desArgSerPhePhe; (D) SAA-2α.

**Figure 7:**
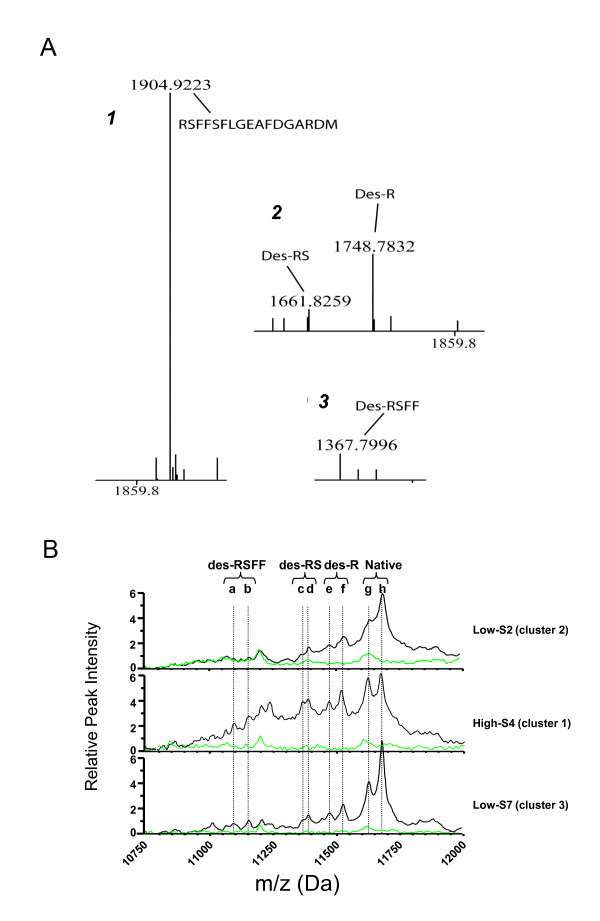
**N-terminal truncated variants of SAA-1α and SAA-2α in HDL**. **A**. Four SAA-1α and SAA-2α isoforms were separated by 2-DE and analyzed by peptide mass fingerprinting after CNBr cleavage. (1) Full-length N-terminal peptide-mass detected in two of the isoforms identified as SAA-1α and SAA-2α; (2, 3) Truncated peptide masses (desArg (des-R), desArgSer (des-RS) and desArgSerPhePhe (des-RSFF)) detected in the two isoforms with an acidic charge shift in 2-DE compared to corresponding native variant (illustrated in figure 6). **B**. Three representative SELDI spectra demonstrating the presence of the SAA isoforms. Depicted are raw spectra of three different individuals before (green lines and 24 hours after (black lines) LPS infusion. The different SAA isoforms are indicated by the subsequent dashed vertical lines; a and b; des-RSFF of SAA-2α and -1α, c and d; des-RS of SAA-2α and -1α; e and f; des-R of SAA-2α and -1α and g and h; Native SAA-2α and -1α, respectively.

**Figure 8:**
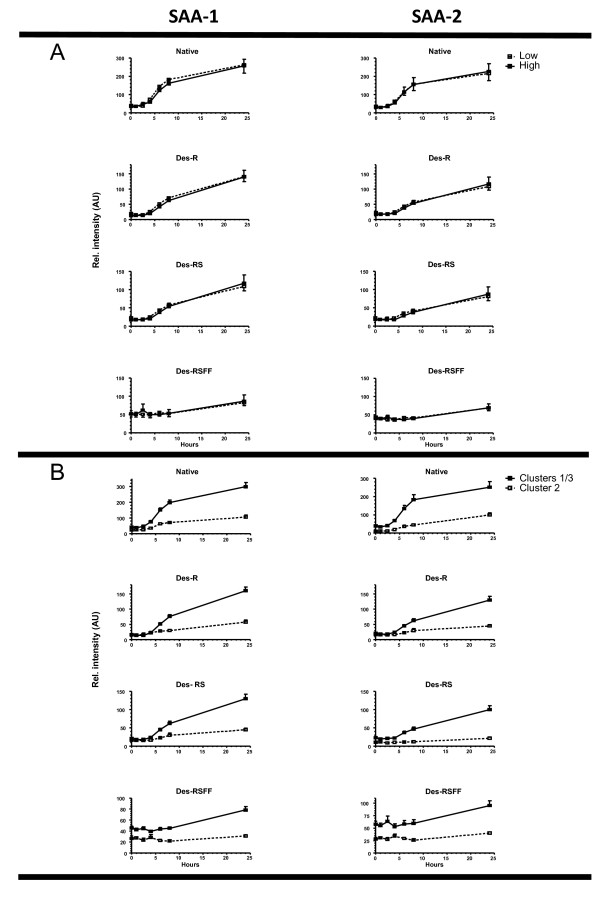
**Dynamics of the SAA-1α and SAA-2α variants**. **A**. The SAA dynamics between the low and high HDL group of SAA-1α (left column) and SAA-2α (right column). **B**. The SAA-1α (left column) and SAA-2α (right column) dynamics in clusters 1/3 (combined) and cluster 2 (p < 0.001). Native SAA and the desArg, desArgSer and desArgSerPhePhe variants are indicated including their corresponding theoretical molecular mass (Daltons).

**Figure 9:**
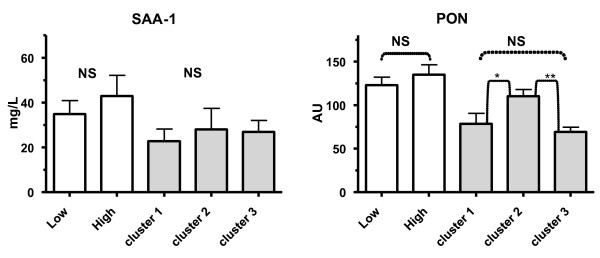
**Comparison of the clusters versus the high and low HDL cholesterol group**. In the panels the overall mean (over time) serum amyloid-A (SAA), paraoxonase (PON) levels are demonstrated of the three calculated clusters (gray bars) and the two HDLc groups (white bars). PON levels showed only a statistical significant difference between the clusters whereas in the SAA levels no statistical significant differences were observed at all. The error bars indicate mean ± SEM. * Indicates a statistical significant difference p < 0.01, ** indicates p < 0.001 and **NS **indicates a non-significant difference.

Given that the molecular masses of SAA-1α and SAA-2α are 11680 and 11630 Da, respectively, the SAA dynamics could also be followed in the SELDI-TOF MS spectra: a clear increase of the signal was observed in the interval between 110027 and 12003 m/z interval representing different SAA-1/2 isoforms (Figure 5). Additional confirmation was achieved by 2-DE, showing a clear upregulation of four SAA-1/2 isoforms after the LPS challenge (median 6-fold increase, p < 0.05) (Figure 6). These isoforms were further characterized by MS analyses. By using a combination of trypsin and CNBr cleavage patterns, 80-90% sequence coverage was obtained. This made it possible to identify native SAA-1α (SAA-1.1) and native SAA-2α (SAA-2.1) as well as three N-terminally truncated variants of each form: SAA-1α-desArg, -desArgSer, -desArgSerPhePhe and SAA-2α-desArg, -desArgSer, -desArgSerPhePhe (Table 2 and figure 7a). These isoforms were also detectable in the SELDI profiles of the study subjects as can be appreciated in three representative examples (one per cluster) (Figure 7b). Detailed SELDI-TOF MS analyses showed that the responses to LPS for these altogether 8 SAA-1/2 forms, with slightly different molecular masses, were not different between the high and the low HDL-C groups (Figure 8). However, the individuals in cluster 2 demonstrated a much lower increase of SAA-1/2 forms in response to LPS than the other two clusters (Figure 8). The onset of the response was also later (> 8 h after LPS exposure) in particular for the truncated variants. Interestingly, the most truncated variants (SAA-1α- and SAA2α-desArgSerPhePhe) had late responses in all individuals but the levels were lower in cluster 2 already before LPS exposure. In Figure 9, in which the mean quantitative SAA-1/2α data over the entire time course are given, no cluster dependent differences could be observed.

**Table 2:** SAA-1 and SAA-2 variants identified in HDL with 2-DE/MS

SAA-1α	Theoretical M*_r _*/pI	SAA-2α	Theoretical M*_r _*/pI
native	11 680/5.9	native	11 630/8.3

desArg	11 530/5.5	desArg	11 470/6.8

desArgSer	11 440/5.5	desArgSer	11 380/7.0

desArgSerPhePhe	11 140/5.5	desArgSerPhePhe	11 090/6.8

As for SAA-1/2, PON1 levels were also similar in the low and high HDL groups (Figure 9). Surprisingly, PON1 levels showed a distinct cluster dependent distribution. Individuals in cluster 2 were associated with the higher PON1 levels compared to the clusters 1 and 3 (p < 0.01 and < 0.001 respectively).

## Discussion

We previously showed that infusion of endotoxin at 1 ng/kg bodyweight had different effects in healthy volunteers with low and high HDL cholesterol levels [12]. Specifically, we reported that the inflammatory response (tumor necrosis factor-alpha, IL-1beta, IL-6, IL-8, and monocyte chemoattractant protein-1) as well as thrombin generation (prothrombin fragment F_1+2_) were significantly increased in the low HDL group. In the current study, we hypothesized whether this discrepancy could be explained by differences in the HDL proteome between the two study groups since many proteins and enzymes that are associated with HDL play a role in inflammation and thrombosis [10,11]. Using HDL immunocapture SELDI-TOF MS, the current analysis shows that the HDL proteome at baseline as well as the dynamic changes in the HDL proteome following LPS administration were, however, not different in the low and high HDL group.

### HDL proteome is not different in individuals with low or high HDL cholesterol as assessed by SELDI-TOF MS

Using a standardized LPS challenge protocol that does not affect HDL cholesterol and apo A-I protein levels [12], 10 individuals with HDL cholesterol levels below the 5^th ^percentile were compared with 10 individuals with HDL cholesterol levels above the 95^th ^percentile. Following endotoxin administration, 104 marker-intervals showed a raw p-value lower than 0.05 and 21 remained significant after correction for multiple comparisons. Most of these markers were found in the lower m/z range of the HDL protein mass spectrum. Since we previously identified significant differences between the systemic inflammatory responses in the high and low HDL groups, we expected to find differences in HDL associated proteins at baseline and/or an effect on HDL proteome dynamics, as readout of the differences in the cytokine storm that was elicited by the intravenous LPS administration. Unexpectedly, heatmaps showed that the HDL protein dynamics were not different amongst the high and low HDL groups at baseline and over the time course. These data thus suggest that the differences in HDL proteome in subjects with low and high HDL do not explain the differences in inflammatory and pro-thrombotic dynamics among these groups.

### Identification of subgroups with different HDL proteome dynamics

Using a bioinformatics approach (on the entire dataset), we identified 3 distinct clusters in HDL proteome dynamics. In line with our initial analysis, this clustering proved largely independent of baseline HDL cholesterol levels in 2 out of the 3 clusters which comprised 80% of all study participants. This analysis confirms that HDL cholesterol levels per se cannot explain why HDL is associated with a difference in response to a LPS challenge. This result is in line with the concept that HDL function parameters should be considered rather then HDL cholesterol concentration when it comes to the protective properties that are attributed to HDL [15]. In this regard, both bioactive lipids as well as proteins are thought to mediate specific functions of HDL which is further supported by findings that HDL subclasses are described to have different properties [11,16,17].

### SAA-1/2 and PON1

With the definition of 3 subgroups, we studied 2 parameters in more detail, SAA-1/2 and PON1.

Serum amyloid-1/2 (SAA-1/2), an acute phase protein, is known to be primarily associated with HDL [18,19]. In addition, our SELDI TOF MS data showed a clearly increased signal in the SAA-1/2 marker interval indicating that this SAA-1/2 was indeed associated with HDL. The masses which correspond with the SAA variants appear also in the top 10 ranking of the found relevant markers which changed upon LPS challenge thereby confirming that SAA is one of the proteins having a major contribution to the cluster classification. SAA association with HDL was confirmed by 2-D gel electrophoresis of the HDL proteome after isolation of HDL by ultracentrifugation. The 2-DE/MS approach also verified the presence of both SAA-1α and SAA-2α in the HDL samples both as native and N-terminally truncated variants. SAA-1α and SAA-2α have over 90% sequence identity. Based on charge and/or molecular mass differences, the combination of 2-DE/MS and SELDI-TOF MS made it possible to identify and measure 8 forms of SAA-1/2. This is in accordance with previous findings which report the presence of SAA-1/2 isoforms caused by post-translational modifications [20,21]. Interestingly, our results showed that all individuals in cluster 2 had a much lower response for the SAA-1/2 isoforms after LPS exposure. The truncated variants of cluster 2 had a delayed response in comparison to the native forms. In addition, the subjects in cluster 2 had lower levels of the most truncated variant (des-ArgSerPhePhe) already before LPS exposure. Although truncated forms of SAA-1/2 have been described before, it is still unclear how these modifications may affect the function. However, our findings are in line with results showing delayed serum response of truncated SAA-1/2 isoforms in an individual with acute inflammation [22]. It would have been interesting to investigate the SAA dynamics for more than 24 hours. Unfortunately, there were no samples available of timepoints after 24 h.

PON1 is an enzyme with strong anti-oxidative properties that is primarily associated with HDL, [23,24] but its role in cardiovascular disease is not undisputed [17,25,26]. Although we could observe changes in intensity in m/z corresponding with the PON-1 mass region, direct identification of PON1 in the individual SELDI spectra was not feasible in the current study. Direct measurement of PON activity however, revealed cluster associated differences in 2 of the 3 clusters. The individuals in cluster 2, presented the highest PON1 activity. In the 2 other clusters, PON1 activities were similar. Surprisingly all clusters contained individuals having both high and low HDL cholesterol levels. It can not be excluded that the found cluster dependent PON1 activity association may be due to genotype dependent differences in PON1 activity response [26].

Our data suggest that the differences in SAA-1/2 isoforms and PON1 in HDL are associated with a differential response to LPS challenge independent of HDL cholesterol levels. However, the significance of these findings and the underlying biological mechanisms remain to be investigated.

### Considerations

Generation of reliable HDL protein profiling data requires careful and routine plasma collection and strict on-chip capture conditions. The protocol used in this study has already been reported to result in low on-chip variability in saturation of HDL capture [9]. Based on the virtually unchanged apo A-I and HDL cholesterol levels (changes were less then 10%, p-value not statistical different for both parameters) over time it was valid to assume that the total particle number did not change to a great extent in this study (Additional file 1, Figure S1).

In this study, we have captured HDL directly from plasma with antibodies against apo A-I without any pretreatment other than freezing plasma at -80 degrees Celsius prior to use. This procedure is therefore anticipated to not largely perturb the HDL proteome. A potential limitation in this study is that no antibodies against apo A-II were used for capturing this specific population of HDL particles not containing apo A-I. Through performing infusions with vehicle in 4 individuals, it is furthermore safe to state that diurnal effects did not contribute to the observed HDL protein dynamics but were solely the result of the endotoxin challenge. Despite the low number of study participants we here show that the semi-quantitative SELDI-TOF MS data, can be used as a contributing tool to study complex biological mechanisms such as the response of humans to an acute inflammatory challenge. However, other tools are needed to identify the proteins in marker regions and how they affect physiology.

## Conclusions

In our initial, study we have shown that subjects with low HDL cholesterol were more susceptible to an inflammatory challenge compared to subjects with high HDL cholesterol levels [12]. The current SELDI-TOF MS study shows that differences in the HDL proteome between these groups cannot explain this finding. This may indicate that the susceptibility for an acute inflammatory challenge may be intrinsic to the HDL particle, and not to the associated proteins on HDL. Analyzing the data of all study participants, we identified three distinct clusters of individuals with distinctly different HDL proteome dynamics of which SAA (isoforms) had a major contribution. Further analyses revealed that PON1 activity levels were associated with HDL proteome changes following LPS challenge.

## Competing interests

The authors declare that they have no competing interests.

## Authors' contributions

JHML designed and carried out the SELDI-TOF and additional experiments. HK, SL and LF carried out the 2D electrophoresis experiments. PG, RM and LW were responsible for the bioinformatics. ML, ESGS, JAK and JCMM were responsible for the design of the study. All authors were involved in writing the manuscript, and have read and approved the final manuscript.

## Supplementary Material

Additional file 1 **Figure S1. HDL cholesterol and apo A-I dynamics**. Serial change of the HDL cholesterol (upper panel) and Apo A-I lower (panel) levels after LPS infusion (1 ng/kg body weight) of the low and high HDL cholesterol group. Virtually no change in levels was observed over the 24 hrs time span (P = 0.99 and 0.94 for HDL cholesterol low and high respectively, P = 0.81 and 0.14 for apo A-I low and high respectively) Click here for file

Additional file 2 **Table S1. Overview of all relevant m/z marker-intervals upon LPS challenge**. The m/z marker intervals with the associated p-values and adjusted p-values for multiple measurements of the LPS treated groups (LPS, n = 20) and the p-values of the healthy subjects (Controls, n = 4) respectively. The marker intervals of the LPS treated group are ordered according to increasing p-value. The corresponding marker intervals in the controls show no p-value lower than 0.05 among all the m/z intervals. Click here for file
