# Supplementary figures and images for: High-density lipoprotein proteome dynamics in human endotoxemia
Source: Proteome Sci. 2011 Jun 28;9:34. doi: 10.1186/1477-5956-9-34 (PMC3146904; doi:10.1186/1477-5956-9-34)

## Slide 1
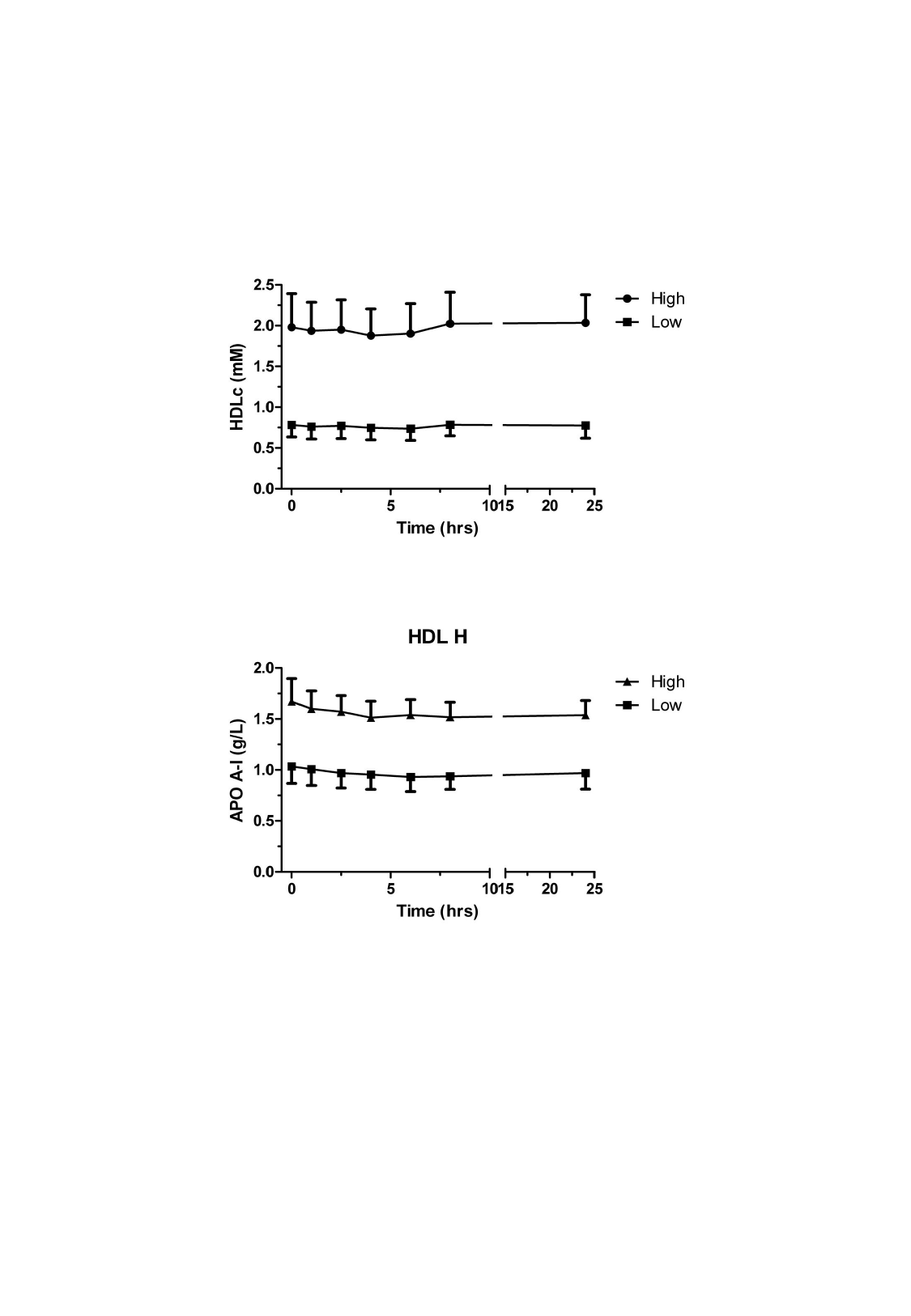

Supplement: Additional file 1 — Figure S1. HDL cholesterol and apo A-I dynamics. Serial change of the HDL cholesterol (upper panel) and Apo A-I lower (panel) levels after LPS infusion (1 ng/kg body weight) of the low and high HDL cholesterol group. Virtually no change in levels was observed over the 24 hrs time span (P = 0.99 and 0.94 for HDL cholesterol low and high respectively, P = 0.81 and 0.14 for apo A-I low and high respectively) [file 1477-5956-9-34-S1.PPT]
