# Supplementary material for: High-density lipoprotein proteome dynamics in human endotoxemia
Source: Proteome Sci. 2011 Jun 28;9:34. doi: 10.1186/1477-5956-9-34 (PMC3146904; doi:10.1186/1477-5956-9-34)
Supplement: Supplementary file 2 [file 1477-5956-9-34-S2.DOC]

Rank	Attribute	P-value (LPS)	Adj. p-value (LPS)	p-value controls
1	M11766-11825	0,000	0,000	0,441
2	M11707-11766	0,000	0,000	0,392
3	M11649-11707	0,000	0,000	0,451
4	M11825-11884	0,000	0,000	0,252
5	M11476-11533	0,000	0,000	0,380
6	M5853-5882	0,000	0,000	0,098
7	M11533-11591	0,000	0,000	0,413
8	M11884-11943	0,000	0,000	0,366
9	M11591-11649	0,000	0,000	0,429
10	M11419-11476	0,000	0,000	0,339
11	M5824-5853	0,000	0,000	0,118
12	M11943-12003	0,000	0,002	0,346
13	M5795-5824	0,000	0,003	0,105
14	M11362-11419	0,000	0,007	0,264
15	M5766-5795	0,000	0,007	0,099
16	M11306-11362	0,000	0,011	0,265
17	M11249-11306	0,001	0,021	0,237
18	M5737-5766	0,001	0,021	0,115
19	M3484-3502	0,001	0,025	0,437
20	M5882-5912	0,001	0,027	0,100
21	M3467-3484	0,001	0,032	0,443
22	M3811-3831	0,002	0,076	0,211
23	M3000-3015	0,004	0,118	0,157
24	M3015-3030	0,005	0,135	0,159
25	M11082-11138	0,005	0,151	0,231
26	M11193-11249	0,006	0,155	0,262
27	M5709-5737	0,006	0,155	0,101
28	M3432-3450	0,007	0,171	0,416
29	M11027-11082	0,007	0,171	0,136
30	M3030-3045	0,008	0,176	0,165
31	M3045-3060	0,008	0,176	0,144
32	M7777-7816	0,009	0,198	0,300
33	M3502-3519	0,010	0,212	0,394
34	M5912-5941	0,010	0,212	0,124
35	M3060-3076	0,011	0,218	0,159
36	M13395-13462	0,011	0,218	0,210
37	M5941-5971	0,012	0,218	0,144
38	M3076-3091	0,012	0,224	0,183
39	M3450-3467	0,013	0,231	0,294
40	M35781-35960	0,013	0,231	0,336
41	M5116-5141	0,014	0,247	0,159
42	M4915-4940	0,015	0,247	0,175
43	M5680-5709	0,015	0,252	0,085
44	M4891-4915	0,017	0,269	0,148
45	M11138-11193	0,017	0,269	0,228
46	M3662-3681	0,018	0,273	0,155
47	M36140-36321	0,020	0,301	0,222
48	M7855-7895	0,021	0,301	0,323
49	M7816-7855	0,022	0,301	0,235
50	M3091-3107	0,022	0,301	0,149
51	M35960-36140	0,022	0,301	0,300
52	M15174-15250	0,023	0,301	0,322
53	M7974-8014	0,023	0,301	0,186
54	M8466-8508	0,023	0,301	0,393
55	M7739-7777	0,024	0,301	0,306
56	M35426-35603	0,024	0,302	0,464
57	M4561-4584	0,025	0,308	0,290
58	M35250-35426	0,026	0,308	0,420
59	M4940-4965	0,027	0,308	0,171
60	M3415-3432	0,027	0,308	0,278
61	M35603-35781	0,027	0,308	0,376
62	M3793-3811	0,028	0,308	0,425
63	M3681-3699	0,028	0,308	0,207
64	M4965-4990	0,029	0,308	0,223
65	M5596-5624	0,031	0,308	0,083
66	M3107-3122	0,031	0,308	0,179
67	M4538-4561	0,031	0,308	0,319
68	M3122-3138	0,031	0,308	0,149
69	M5624-5652	0,032	0,308	0,102
70	M5568-5596	0,033	0,308	0,099
71	M4867-4891	0,033	0,308	0,189
72	M5513-5540	0,034	0,308	0,138
73	M6597-6630	0,034	0,308	0,240
74	M8723-8766	0,035	0,308	0,334
75	M3831-3850	0,035	0,308	0,188
76	M13328-13395	0,035	0,308	0,185
77	M7934-7974	0,035	0,308	0,228
78	M8135-8175	0,035	0,308	0,231
79	M5652-5680	0,036	0,308	0,100
80	M3927-3947	0,036	0,308	0,267
81	M7700-7739	0,037	0,308	0,235
82	M34040-34210	0,038	0,308	0,233
83	M46145-46376	0,038	0,308	0,278
84	M4990-5014	0,038	0,308	0,211
85	M9078-9123	0,038	0,308	0,325
86	M45915-46145	0,038	0,308	0,313
87	M13130-13196	0,038	0,308	0,177
88	M13529-13597	0,039	0,308	0,169
89	M5540-5568	0,039	0,308	0,125
90	M8766-8810	0,040	0,308	0,244
91	M13196-13262	0,040	0,308	0,159
92	M3519-3537	0,041	0,308	0,338
93	M6564-6597	0,041	0,308	0,246
94	M5971-6001	0,043	0,321	0,160
95	M35074-35250	0,043	0,321	0,503
96	M34210-34382	0,045	0,327	0,394
97	M34382-34553	0,046	0,329	0,355
98	M4842-4867	0,046	0,330	0,163
99	M4630-4653	0,047	0,330	0,173
100	M15950-16029	0,048	0,330	0,222
101	M46607-46840	0,049	0,330	0,260
102	M4676-4700	0,050	0,330	0,248
103	M47075-47310	0,050	0,330	0,171
104	M12123-12184	0,050	0,330	0,249
